# Eczematous eruption during bimekizumab treatment in a psoriatic patient previously treated with secukinumab

**DOI:** 10.1002/ski2.371

**Published:** 2024-08-29

**Authors:** Gennaro Marco Falco, Giacomo Caldarola, Alessandra D’Amore, Lorenzo Maria Pinto, Clara De Simone, Ketty Peris

**Affiliations:** ^1^ Dermatologia Dipartimento di Medicina e Chirurgia Traslazionale Università Cattolica del Sacro Cuore Rome Italy; ^2^ UOC di Dermatologia Dipartimento di Scienze Mediche e Chirurgiche Fondazione Policlinico Universitario A. Gemelli – IRCCS Rome Italy

## Abstract

Several eczematous eruptions have been described during treatment with anti‐IL17A and anti‐IL17 receptor drugs. In our case, however, the patient had been treated for 2 years with an IL‐17A inhibitor without ever developing eczematous reactions, which occurred, however, shortly after starting therapy with bimekizumab, an IL‐17A, F and A/F inhibitor.

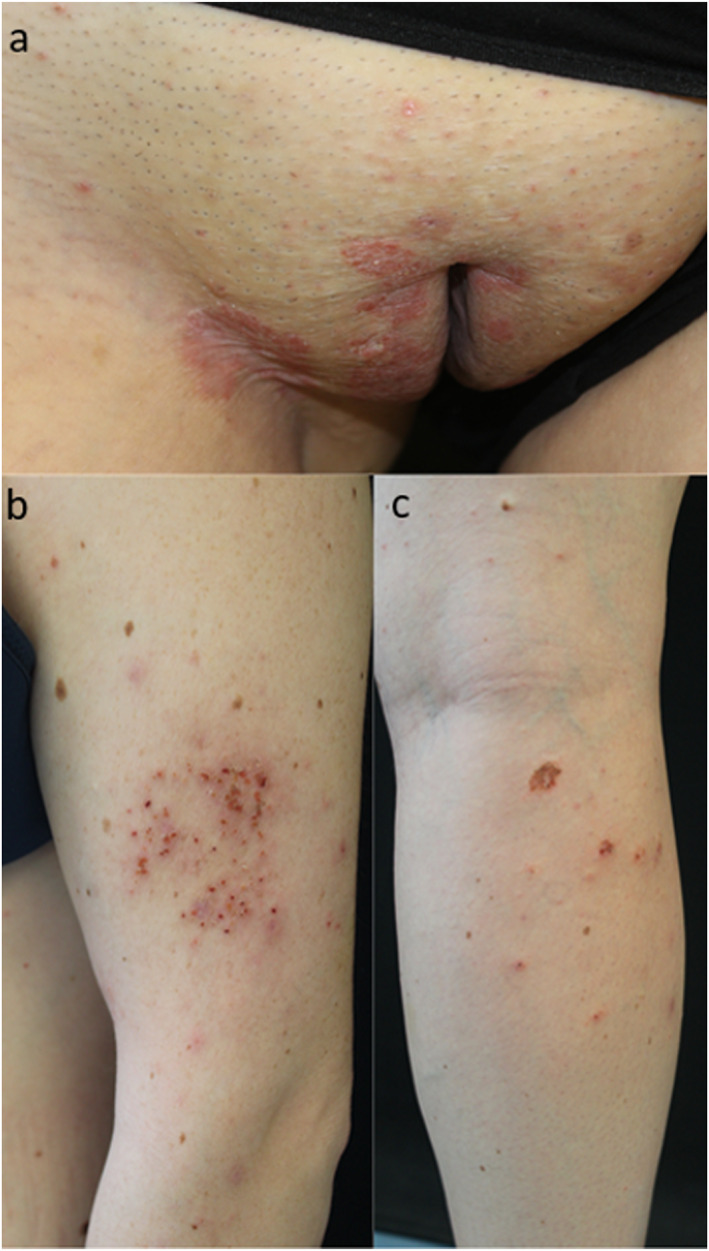

Dear Editor,

A 27‐year‐old woman, with plaque psoriasis since the age of 14, was examined for the appearance of an eczematous eruption, occurring during therapy with bimekizumab. After discontinuing previous therapy with Cyclosporin 3 mg/kg for increased blood pressure, the patient had been treated for 2 years with secukinumab, which was then discontinued due to complete remission obtained during the first month of therapy. Recurrence of psoriatic plaques on the groin (Figure 1a), submammary folds and scalp (PASI 8) was observed 6 months after secukinumab interruption and, in accordance with patient, a treatment with bimekizumab was started at scheduled dosages of 320 mg by subcutaneous injection every 4 weeks for 16 weeks and every 8 weeks thereafter with the aim to reduce the number of drug injections compared to the previous treatment.

**FIGURE 1:**
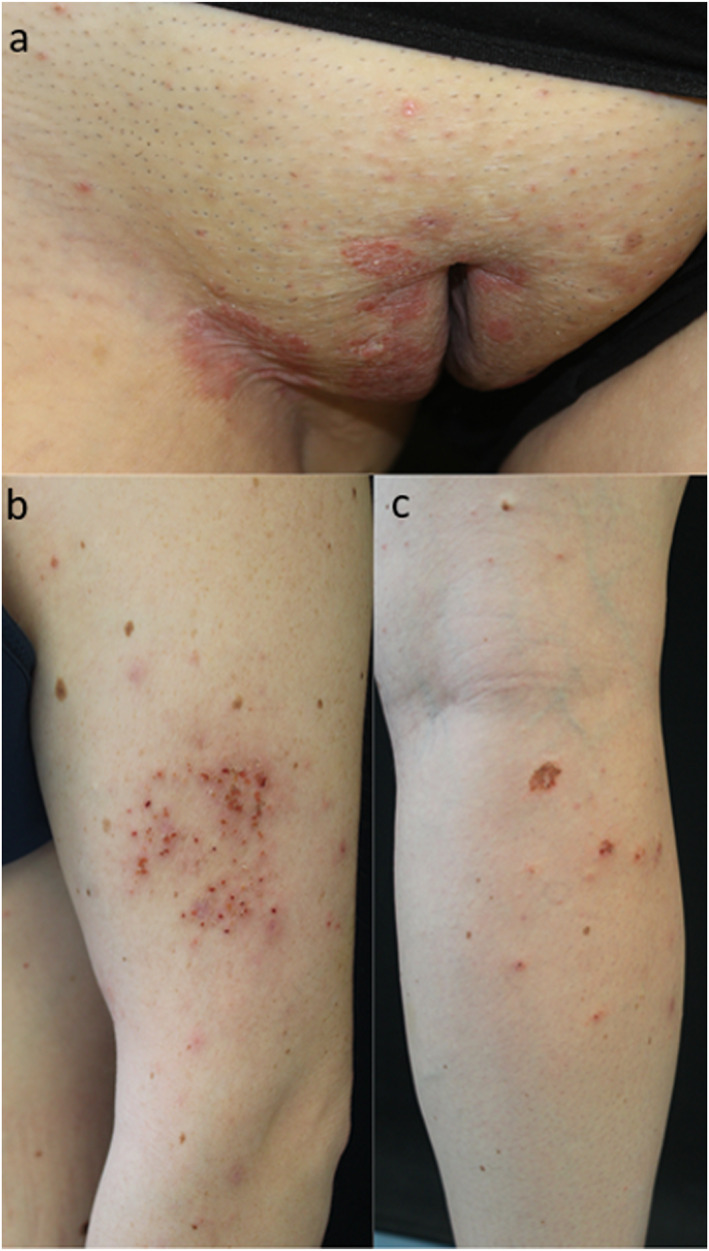
(a) Multiple psoriasis plaques in the groin. (b) Single eczematous patch localised on the left arm, appeared 1 month after starting bimekizumab. (c) Multiple eczematous lesions localised on the right leg, appeared 2 months after starting bimekizumab.

One month after treatment initiation with bimekizumab, complete clearance of psoriasis (PASI 0) was achieved but at the same time an erythematous patch, with poorly defined borders and covered with excoriations appeared on the left arm (Figure 1b). Epicutaneous patch tests were consequently performed and were negative. A punch biopsy was performed on the eczematous lesion and histopathology showed the presence of severe spongiosis with spongiotic vesicles and lymphocitic exocytosis (Figure 2). Therefore, a medium‐potency topical steroid therapy was initiated, while bimekizumab was continued. After 2 months of treatment, the eczematous lesion on the arm was persistent and additional lesions appeared on the lower limbs (Figure 1c). Then, bimekizumab was discontinued and complete regression of cutaneous lesions was obtained with a 15‐day course of systemic steroids.

**FIGURE 2:**
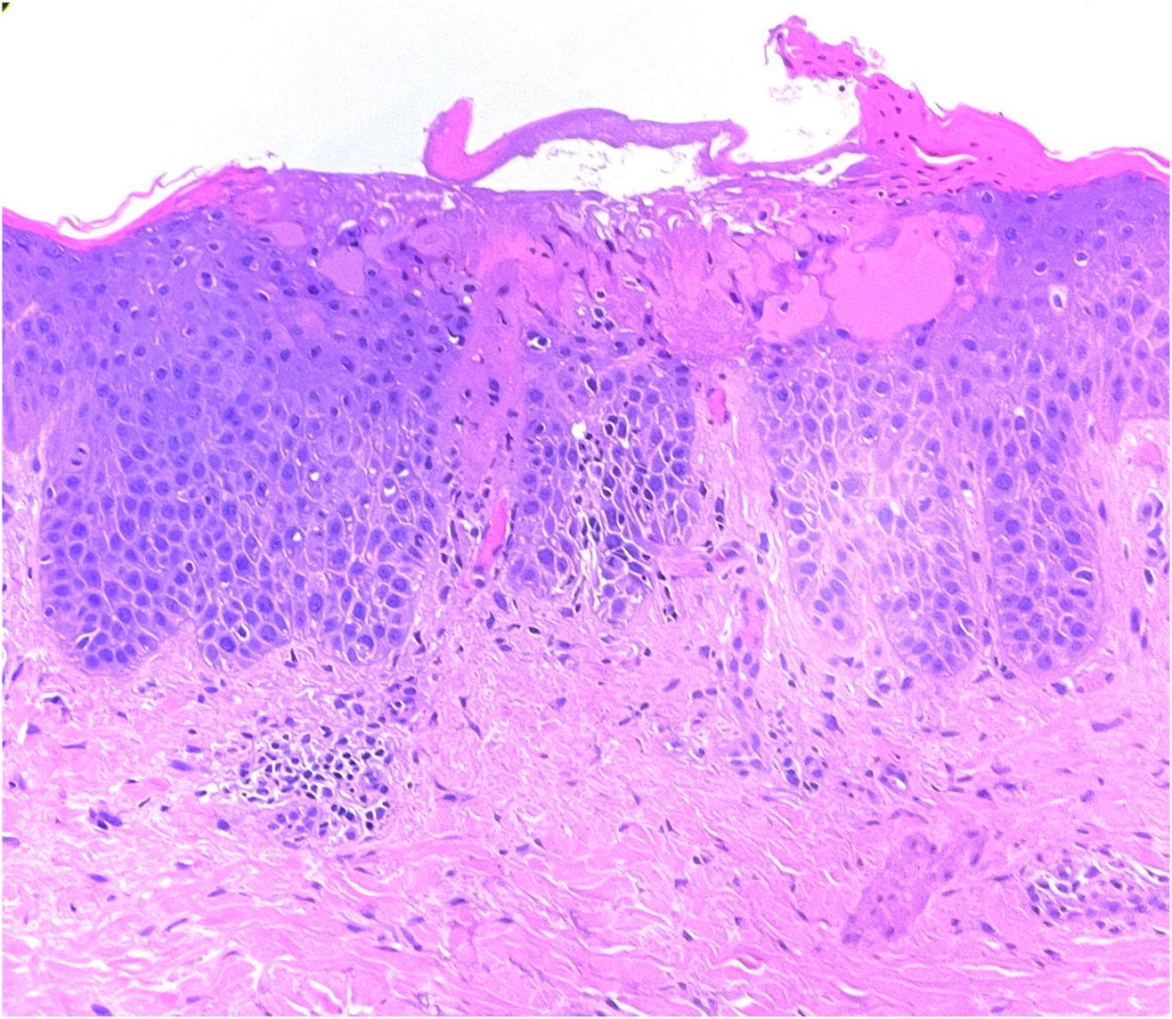
Histopathology of an eczematous lesion showing severe spongiosis with spongiotic vesicles and lymphocitic exocytosis.

Bimekizumab is a recently approved humanised monoclonal IgG1 antibody that targets interleukin (IL)‐17A, IL‐17F and IL‐17A/F, showing high level of efficacy in psoriatic patients and a good safety profile.^1^ To date, several eczematous eruptions have been described during treatment with anti‐IL17A and anti‐IL17 receptor drugs, such as secukinumab, ixekizumab and brodalumab.^2^ The clinical presentation can range from classical acute and psoriasiform eczema to atopic dermatitis‐like eruption, that share the presence of spongiosis on histopathological examination.^3^

The pathogenesis of eczematous reactions during therapy with anti‐IL17 agents is not yet well defined. It has been hypothesised that blockade of the Th1/Th17 axis may result in activation of the Th2 axis, thus leading to the occurrence of eczemas.^4^ In particular, IL‐4 and IL‐22 seem to be the main cytokines of the Th2 axis involved in the pathogenesis of these reactions as demonstrated in a previous study that assessed the cytokine gene expression of patients who developed anti‐IL17A induced eczemas.^5^ Another hypothesis suggests that blocking IL‐17A may induce increased levels of another pro‐inflammatory IL‐17 isoform, such as IL‐17C. IL‐17C is a cytokine of keratinocyte origin believed to be involved in the pathogenesis of both psoriasis and atopic eczema: in particular it stimulates an autocrine loop mediated by TNFalpha and involving both Th17 and Th2 axis cytokines.^6^, ^7^ This hypothesis is in line with the low rate of eczematous eruptions reported with the anti‐ IL17 receptor agent, brodalumab, which blocks all the IL‐17 isoforms. In our case, the blockade of IL‐17A alone mediated by secukinumab, which had been administered in the prior 2 years, may not have been sufficient to trigger an eczematous reaction in our patient, which may have been triggered instead by the simultaneous blockade of IL17A and IL17F mediated by bimekizumab.

To our knowledge, this is the first case of an eczematous eruption in one patient treated with bimekizumab. Further studies on a larger scale are needed to evaluate if blocking IL‐17A and IL‐17F isoforms may be associated to a higher rate of this adverse event than blocking exclusively the IL‐17A.

## CONFLICT OF INTEREST STATEMENT

G. Caldarola has received consulting fees, honoraria and support for attending meetings from Abbvie, Lilly, Janssen, UCB, Novartis and Leopharma; C. De Simone has received support for consulting fees, honoraria and support for attending meetings from Abbvie, Lilly, Janssen, UCB, Novartis, Leopharma, Sanofi and Almiral; K. Peris has received support for consulting fees and honoraria from Abbvie, Almirall, Biogen, Celgene, Janssen Galderma, Novartis, Lilly, Novartis, Pierre Fabre, Sandoz, Sanofi and Sun Pharma. All other authors declare that they have no conflicts of interest relevant to this manuscript.

## AUTHOR CONTRIBUTIONS

**Gennaro Marco Falco**: Writing—original draft (lead). **Giacomo Caldarola**: Writing—review and editing (lead). **Alessandra D’Amore**: Supervision (equal). **Lorenzo Maria Pinto**: Writing—original draft (supporting). **Clara De Simone**: Supervision (supporting). **Ketty Peris**: Supervision (lead).

## FUNDING INFORMATION

This article received no specific grant from any funding agency in the public, commercial, or not‐for‐profit sectors.

## ETHICS STATEMENT

The patients in this manuscript have given written informed consent to publication of their case details.

## Data Availability

Data sharing is not applicable to this article as no new data were created or analyzed in this study.
